# Undergraduate Training in Public Health Should Prepare Graduates for the Workforce

**DOI:** 10.3389/fpubh.2014.00285

**Published:** 2015-01-05

**Authors:** Randy Wykoff, Amal Khoury, J. Michael Stoots, Robert Pack

**Affiliations:** ^1^Department of Health Services Management and Policy, College of Public Health, East Tennessee State University, Johnson City, TN, USA; ^2^Department of Community and Behavioral Health, College of Public Health, East Tennessee State University, Johnson City, TN, USA

**Keywords:** undergraduate public health, BSPH, workforce preparation, public health job market, college of public health, benchmarking

There has been a rapid growth in the number of programs awarding undergraduate degrees in public health and the number of students receiving such degrees (1). There has not, however, been a significant discussion of the purpose of such degree programs. What, if anything, are the recipients of these degrees being trained to do? What careers, if any, are they being prepared to enter? Is the degree designed primarily to prepare students to enter graduate training in public health or some other graduate or health professional programs? Alternatively, does the degree exist because “an understanding of public health is a critical component of good citizenship and a prerequisite for taking responsibility for building healthy societies”? (2).

While a reasonable case could be made for all of these purposes, we strongly believe that the undergraduate degree in public health should be seen primarily as a professional degree that is designed to prepare students to enter a well-defined and vital career track.

Our perspective is informed by a 60-year history of providing undergraduate training in public health and by a long-standing relationship with alumni, preceptors, and employers who regularly provide quantitative survey data to us. While our specific experience reflects our long history and our geographic location in a relatively rural area of central Appalachia, we believe that our “lessons learned” are relevant to any program currently or potentially providing undergraduate education in public health.

Specifically, it is helpful for any program to seek to understand its market and its students; tailor its competencies and curriculum to match the needs of its employers; and continuously evaluate its performance by seeking input from students, alumni, employers, and other stakeholders. As seen below, those processes and self-assessment tools have been key to the success and longevity of our program.

East Tennessee State University (ETSU) has been offering undergraduate training in public health for well over 60 years. In 1933, the State Teacher’s College, Johnson City (the predecessor of ETSU) began offering a concentration in Health within the degree that was then known as Physical Education and Health. A minor in Health was first offered in 1950, and the School of Health was created in 1955. It included the newly formed Department of Health Education^1^ and offered, for the first time, a BS in Health Education. The Department of Environmental Health was created and first offered the Bachelor of Science in Environmental Health (BSEH) in 1965. The Bachelor of Science in Health Science (BSHS) was also first offered in 1965. In 1969, the BSEH became the first undergraduate program in the United States accredited by the National Accreditation Council for Undergraduate Curricula in Environmental Health. In 1973, a concentration in Health Administration was offered by the Department of Health Education and, in 1978, the School of Public and Allied Health^2^ was established. Masters degrees were added in Environmental Health (MSEH in 1971) and Public Health (MPH in 1986). In 2000, ETSU was accredited by the Council on Education for Public Health (CEPH) as a graduate program in public health and, in 2009, was accredited as the first school of public health in Tennessee and the first to be located in central Appalachia. By definition, with full accreditation as a school of public health, all academic programs in the college, including the undergraduate degrees, were part of the CEPH-accredited unit. This chronology makes ETSU one of the few accredited schools of public health in the country that started with undergraduate training and added graduate programs at a later date.

Today, the ETSU College of Public Health, in addition to a full complement of graduate degrees and certificates, offers five undergraduate degrees – BS Public Health (BSPH) with concentrations in Community Health and Health Care Administration; BSHS with concentrations in Microbiology and Human Health; and the BSEH. In the 25 years between 1989 and 2014, ETSU awarded over 1,500 undergraduate degrees from what became the College of Public Health – 722 BSPH degrees, 530 BSEH degrees and 294 BSHS degrees.

This article addresses recent data generated by the alumni, preceptors, and employers of the BSPH graduates. According to the ASPPH interactive website, in the first 5 years reported (1992–1996), ETSU was the fifth most productive program in the country. In the most recent 5 years (2009–2013), despite having awarded 18% more degrees than in the first 5 years, ETSU’s relative position has dropped to 29th nationally, reflecting the rapid growth in number and size of other programs (3).

In 2009/2010, 2010/2011, and 2011/2012, 65 BSPH graduates have responded to our alumni survey, conducted each year about 18 months after graduation (Table 1).

**Table 1:** **Reported placement data: BSPH alumni survey (2009/2010, 2010/2011, 2011/2012 alumni) (65 responders: response rate 69.5%)**.

Placement	Number	Percent^a^
Hospital or healthcare delivery organization	21	34.4
Non-profit organization	6	9.8
Local, state, or federal government	4	6.6
Proprietary organization	3	4.9
University	2	3.3
School system	2	3.3
Other	10	16.4
Unemployed: looking for work	2	3.3
Unemployed: not looking for work	4	n/a
Student	11	18.0

*^a^Excludes those unemployed: not looking for work*.

It is clear, from these data that our BSPH graduates are most likely to enter the workforce, especially into positions with hospitals and healthcare delivery organizations. They are relatively less likely to enter into the “traditional” public health careers in local, state, and federal government organizations. Only about a fifth directly enter graduate school. This latter finding is consistent with the findings of Leider et al. that report that fewer than 10% of graduates from undergraduate programs in public health apply to medical school or graduate programs in public health.

Results from our alumni survey also indicate that these graduates felt adequately prepared for their careers. Of those 65 BSPH graduates who responded to the 2011, 2012, and 2013 alumni surveys, 97% reported being “very satisfied” or “somewhat satisfied” with their overall academic experience and 95% said that they would recommend the college to others.

Their perspectives are supported by the College’s employer surveys and field preceptor surveys. Every other year, the college surveys employers who report hiring one or more graduates from the College. Of the 107 employers who responded to the 2011 and 2013 surveys (in some cases, the same employers responded to both surveys), 96 employers identified the academic degrees of their recent employees, and of those, 53 (55%) reported hiring one or more BSPH graduates. Because the same employer often hires graduates of multiple degree programs, we are not able to report results specifically for employers of BSPH graduates. However, 102 of 107 (95%) employers ranked the College’s graduates as “high” or “highest” in “overall competence of graduates in their field of practice”; 98% for “ability to understand and use technical information”; 89% in “knowledge of public health”; and 93% reported “likelihood of hiring future College graduates.”

The other source of information suggesting that BSPH graduates are ready to enter the job market comes from the students’ preceptors. Prior to graduation, all BSPH students must complete an internship (culminating experience) that includes at least 400 service-learning hours at a relevant organization, and under the supervision of a qualified preceptor. At the conclusion of each internship, the College formally surveys the students’ preceptors.

In 2011/2012, 2012/2013, and 2013/2014, 155 BSPH students received preceptor evaluations. Using a 5.0 scale, preceptors are asked to evaluate students for a range of concentration-specific competencies (which vary by concentration) and for six cross-cutting competencies (work ethic; reliability; self confidence and interpersonal skills; systems thinking and innovation; inquisitiveness and desire to learn; and ability to manage multiple assignments simultaneously). Over the 3 years, the average score for the cross-cutting competencies was 4.80 and the average score for the concentration-specific competencies was 4.71.

While the curriculum for the BSPH has been recently revised (see accompanying article by Stoots et al.), it retains the key elements that have defined the degree for its more than half century of history – notably its commitment to preparing students to enter the workforce.

## Conclusion

The current shortage of trained professionals in public health has heightened potential interest in undergraduate training for public health.

Data from our program, and recently reported national data, document that graduates from undergraduate programs in public health are overwhelmingly entering the workforce upon graduation. In our experience, most students enter a health-related job in hospitals, medical practices, nursing homes, or other healthcare industries, though the job destinations may vary in other job markets. The students’ own reports, the evaluations from their internship preceptors, and the evaluations from their employers all suggest that these students are well-prepared and successful in meeting the needs of the workforce.

While recognizing that a minority of students do enter graduate school upon graduation (and presumably more do, sometime later) and while recognizing that training in public health is valuable preparation for many career tracks, the fact that the vast majority of undergraduate public health graduates enter the workforce leads, we believe, to three major conclusions:

(1) Undergraduate degree programs in public health should be designed, delivered, modified, and evaluated primarily with the understanding that they are preparing students for the workforce. Specific attention should be given to assuring that students are “exposed to local level public health professionals and/or to agencies that engage in population health practice” (4), and that they acquire the practical and applied skills necessary for success in the workforce. To achieve these ends, we strongly believe that students should complete a substantial internship in the public health workforce prior to graduation.
(2) Undergraduate programs in public health should be carefully and regularly benchmarked against the needs of local employers. This necessitates regular collection and analysis of data from alumni, preceptors, and employers, as well as periodic re-evaluation of future trends and directions in the needs and expectations of employers. We require each of our programs to undergo such a review every 4 years. An additional source of useful data can be collected from students when they return from their internships. We routinely ask these students to identify areas where their preparation could have been improved.
(3) Institutions offering undergraduate degrees in public health also have an obligation to assure that there is a job market for their graduates. As in any other field, an “over supply” of graduates, or the production of graduates who lack the skills necessary for success in the job market, serves neither the best interests of the field nor the best interest of the graduates. New programs should grow slowly, assuring that the supply of graduates does not exceed the “carrying capacity” of the local job market. A close relationship between the schools and local employers is essential in this process.

We believe that to treat the degree as one that provides only a basic level of understanding of the field is to deny students the quality of training that is essential to protect the public’s health in the future. Undergraduate-trained engineers build bridges and buildings. Why would not undergraduate – trained public health professionals have a comparable level of expertise and skill?

For years academia has recognized the Master of Public Health degree as a “professional” degree designed and executed to prepare students to enter the workforce. The undergraduate degree should be seen in no different light.

## Conflict of Interest Statement

The authors declare that the research was conducted in the absence of any commercial or financial relationships that could be construed as a potential conflict of interest.

## Footnotes

^1^The name of this department was changed to Public Health in 1995.

^2^By 2007, Departments in the College of Public and Allied Health that were split off in the forming of the College of Public Health included Dental Hygiene, Radiography, Cardiopulmonary, Speech and Language Pathology, and Physical Therapy.
